# Magnetic resonance imaging and ultrasound examination in preoperative pelvic staging of early‐stage cervical cancer: *post‐hoc* analysis of SENTIX study

**DOI:** 10.1002/uog.29205

**Published:** 2025-03-25

**Authors:** D. Cibula, C. Köhler, J. Jarkovský, R. Kocián, P. Dundr, J. Klát, I. Zapardiel, F. Landoni, F. Frühauf, R. Fischbach, M. Borčinová, D. Fischerová

**Affiliations:** ^1^ Department of Gynecology, Obstetrics and Neonatology, First Faculty of Medicine Charles University and General University Hospital in Prague Prague Czech Republic; ^2^ Department of Gynecology Asklepios Clinic Hamburg Altona Berlin Germany; ^3^ Department of Gynecology DRK Klinik Berlin Westend Berlin Germany; ^4^ Institute of Biostatistics and Analyses, Faculty of Medicine Masaryk University Brno Czech Republic; ^5^ Department of Pathology, First Faculty of Medicine Charles University and General University Hospital Prague Czech Republic; ^6^ University Hospital Ostrava and Faculty of Medicine University of Ostrava Czech Republic; ^7^ Gynecologic Oncology Unit La Paz University Hospital Madrid Spain; ^8^ UNIMIB‐IRCCS‐San Gerardo Monza Italy; ^9^ Department of Radiology Asklepios Clinic Hamburg Altona Berlin Germany

**Keywords:** cervical cancer, clinical staging, FIGO, imaging, MRI, ultrasound

## Abstract

**Objectives:**

SENTIX was a prospective, single‐arm, international multicenter study that evaluated sentinel lymph node biopsy without pelvic lymph node dissection in patients with early‐stage cervical cancer. We aimed to evaluate the concordance between preoperative imaging modalities (magnetic resonance imaging (MRI) and ultrasound) and final pathology in the clinical staging of early‐stage cervical cancer by *post‐hoc* analysis of the SENTIX study data.

**Methods:**

In total, 47 sites across 18 countries participated in the SENTIX study. Patients with Stage IA1/lymphovascular space invasion‐positive to IB2 (International Federation of Gynecology and Obstetrics (FIGO) classification (2018)) cervical cancer, with usual histological types and no suspicious lymph nodes on imaging, were prospectively enrolled between May 2016 and October 2020. Preoperative pelvic clinical staging on either pelvic MRI or ultrasound examination was mandatory. Tumor size discrepancy (< 10 mm *vs* ≥ 10 mm) between imaging and pathology, as well as the negative predictive value (NPV) of MRI and ultrasound for parametrial involvement and lymph node macrometastasis, were analyzed.

**Results:**

Among 690 eligible prospectively enrolled patients, MRI and ultrasound were used as the staging imaging modality in 322 (46.7%) and 298 (43.2%) patients, respectively. A discrepancy of tumor size ≥ 10 mm was reported between ultrasound and final pathology in 39/298 (13.1%) patients and between MRI and pathology in 53/322 (16.5%), with no significant difference in the accuracy of tumor measurement between the two imaging modalities. The NPV of ultrasound in assessing parametrial infiltration and lymph node involvement was 97.0% (95% CI, 0.95–0.99%) and 94.0% (95% CI, 0.91–0.97%), respectively, and that of MRI was 95.3% (95% CI, 0.93–0.98%) and 94.1% (95% CI, 0.92–0.97%), respectively, with no significant differences between the parameters. Ultrasound and MRI were comparable regarding the tumor size measurement (*P* = 0.452), failure to detect parametrial involvement (*P* = 0.624) and failure to detect macrometastases in sentinel lymph node (*P* = 0.876).

**Conclusions:**

Pelvic ultrasound examination and MRI had similar concordance with histology in the assessment of tumor size and of parametrial and lymph node invasion in early‐stage cervical cancer. Ultrasound examination should be considered part of preoperative pelvic clinical staging in early‐stage cervical cancer, especially in limited‐resource regions where MRI is unavailable. © 2025 The Author(s). *Ultrasound in Obstetrics & Gynecology* published by John Wiley & Sons Ltd on behalf of International Society of Ultrasound in Obstetrics and Gynecology.

## INTRODUCTION

Cervical cancer is a malignancy for which incidence is decreasing dramatically in high‐ and middle‐income countries^1^. However, it still remains the second most common malignancy among females worldwide and is the leading cause of death among females of childbearing age in low‐income regions^2^, where the most recent management innovations, such as immunotherapy^3^, robotic surgery^4^ or pathological ultrastaging of sentinel lymph nodes (SLNs)^5^, are unavailable. In resource‐limited regions, reliable triage of patients at the time of diagnosis is therefore even more essential to ensure that the available resources are used efficiently, especially if curative surgery is possible.

Because the initial spread of cervical cancer is almost never hematological but mostly infiltrative or lymphatic^6^, local pelvic staging is a crucial first step in the management. Tumor size, tumor growth outside the cervix and pelvic lymph node involvement are three preoperatively assessed parameters that determine the subsequent clinical management, including selection of the treatment modality, particularly in early stages of the disease^7^, ^8^, ^9^, ^10^.

It is well documented that physical examination provides inaccurate assessment of tumor size and parametrial involvement, and it does not provide any information about the lymph node status^11^. Therefore, the current international guidelines consider an imaging test a mandatory component for patient triage^7^, ^8^. The current reference standard for pelvic clinical staging is magnetic resonance imaging (MRI)^12^, but this is rarely available in regions with the highest burden of cervical cancer^13^.

In 2008, a single‐center cohort study first demonstrated that ultrasound examination offers similar accuracy to MRI for staging early‐stage cervical cancer^14^, a finding later supported by a prospective multicenter study of 182 patients across four European centers^15^. More recently, the SENTinel lymph node biopsy in cervIX cancer (SENTIX) study aimed primarily to confirm the safety of SLN biopsy alone, without systematic pelvic lymphadenectomy^16^.

The aim of this *post‐hoc* analysis of SENTIX study data was to report and compare the concordance of preoperative MRI and ultrasound assessments with the final pathological results for all three clinically most relevant parameters in the preoperative clinical staging of early‐stage cervical cancer: tumor size assessment, parametrial invasion and lymph node involvement. As both imaging tests were performed in almost equal numbers of patients, this was a unique opportunity to compare both imaging modalities for preoperative staging in routine clinical practice.

## METHODS

### Study design and participants

The SENTIX study was a prospective, single‐arm, international multicenter study conducted as an academic (Model A) European Network of Gynaecological Oncological Trial groups (ENGOT) trial (ENGOT‐CX2) and was led by the Central and Eastern European Gynecologic Oncology Group (CEEGOG‐CX1). The selection criteria used to assess the quality of participating sites were published previously^16^, ^17^. The protocol was approved by the institutional review board at the leading institution (General University Hospital, Prague, Czech Republic) in June 2016 and was subsequently approved by the institutional review boards at all participating institutions. All patients signed written informed consent according to institutional and federal guidelines before enrolment. All details regarding the SENTIX protocol were reported by Cibula *et al*.^16^ and are available at www.clinicaltrials.gov (NCT02494063).

**Figure 1:**
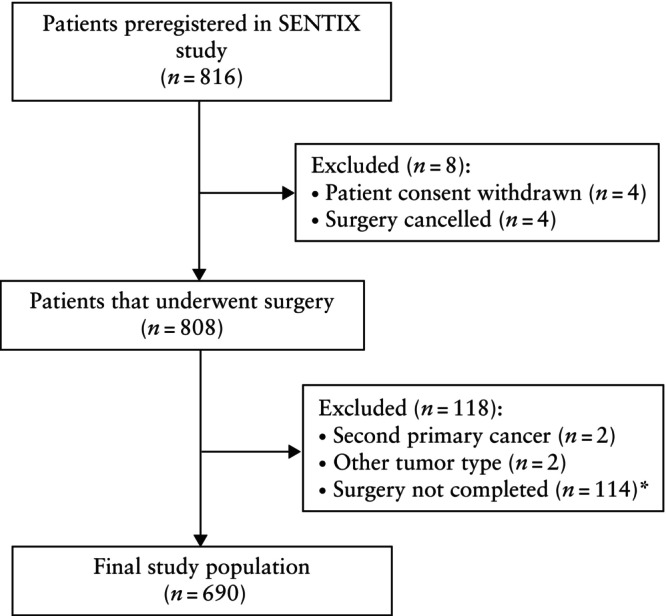
Flowchart showing inclusion in *post‐hoc* analysis of preregistered SENTinel lymph node biopsy in cervIX cancer (SENTIX) study patients. *Due to intraoperative finding of more advanced tumor spread or lymph node involvement.

Patients with early‐stage cervical cancer were enrolled prospectively between May 2016 and October 2020 in the SENTIX study if their disease was Stage IA1 with lymphovascular space invasion, IA2, IB1 or IB2 (International Federation of Gynecology and Obstetrics (FIGO) 2018 classification)^18^, if they lacked suspicious lymph nodes on preoperative imaging, if they had squamous cell carcinoma, adenocarcinoma or adenosquamous carcinoma, and if their largest tumor diameter was < 4 cm or < 2 cm for patients scheduled for a fertility‐sparing procedure. All preregistered patients, in whom surgical treatment was completed, were eligible for this *post‐hoc* SENTIX analysis. Figure 1 summarizes the inclusion of preregistered patients in the analysis.

### Preoperative staging procedures

Physical examination and an imaging test (MRI or ultrasound) for pelvic clinical staging were mandatory in preoperative clinical staging. An invasive cancer diagnosis was confirmed preoperatively on pathology using a specimen obtained by biopsy, excision or any conization technique.

The imaging report was accompanied by a checklist containing all relevant markers for clinical staging, including the largest observed tumor size in mm and its relationship to surrounding structures (i.e. present/absent parametrial involvement, vaginal involvement and lymph node involvement).

The imaging modality used was at the investigator's discretion, so each site used the method with which they were most familiar or was most suitable for the individual patient. At each site, sonographers and radiologists with experience in gynecological oncology performed the respective examinations.

#### Ultrasound imaging

A standardized ultrasound examination was performed by an experienced examiner (Level 3, European Federation of Societies for Ultrasound in Medicine and Biology)^19^. A combination of an endoluminal high‐resolution probe at 5–9 MHz inserted transvaginally or transrectally and transabdominal scanning using a convex array probe at 2–9 MHz was used. The minimum technical requirements included advanced algorithms that allowed investigation with high‐frequency transvaginal and transabdominal probes at greater tissue depths (such as pulse inversion or coded excitation). These algorithms are common in the higher category and are already available in the intermediate category of ultrasound machines. All examiners were advised on the recommended literature source for how to assess the local spread as well as the lymph nodes in cervical cancer^20^, and were also continuously advised by study management to follow the latest updates of the recommendation as available^21^.

#### Magnetic resonance imaging

Pelvic MRI was performed according to the institutional standards with minimum criteria including: (i) 1.5‐ or 3‐Tesla field strength with a pelvic phased‐array coil; (ii) T2‐weighted sequences in sagittal, coronal and axial planes (the axial oblique plane perpendicular to the long axis of the cervix), T1‐weighted sequence in the axial plane, diffusion‐weighted imaging in the axial or oblique axial plane; and (iii) slice thickness of ≤ 4.0 mm with no gap or a minimal gap of ≤ 1.0 mm, field of view 180–240 mm and matrix size 256 × 256 mm. All examiners were advised on the recommended literature source for how to assess the local spread as well as the lymph nodes in cervical cancer and were also continuously advised by study management to follow the latest updates of the recommendation as available^22^.

### Surgical procedures and pathological examination

All patients underwent SLN biopsy, followed by hysterectomy/trachelectomy (radical or simple) or conization. The protocol for SLN detection was as published previously^17^. The type of radical hysterectomy performed (with parametrectomy type B, C1 or C2) was at the discretion of the attending surgeon, based on patient prognostic risk factors^9^.

Patients in whom SLN was not detected or was only detected unilaterally and patients with intraoperative detection of a more advanced disease stage (> IB2)^18^ were excluded from the study after surgery and were subsequently managed according to institutional guidelines.

At each center, a dedicated pathologist with substantial experience in gynecological oncology assessed the pathological specimens. The description of the pathological assessment was defined previously^23^. The comprehensive pathological report was accompanied by a checklist summarizing all relevant markers, including final TNM stage, histological type based on the 2014 World Health Organization classification, tumor size in mm in three dimensions (two measurements of horizontal dimensions and the depth of invasion), relationship to surrounding structures (parametrial involvement (including depth of invasion in mm), vaginal involvement (present/absent) and involvement of surgical margins (both parametrial and vaginal, including distance to the surgical margin)) and presence/absence of lymphovascular space invasion. After intraoperative processing by frozen section, all SLNs were sent for ultrastaging^5^. The quality of pathological processing of SLNs was assessed centrally, as previously reported^24^. Based on the FIGO guidelines^18^, N1 stage was based on the extent of lymph node involvement, as micrometastatic (MIC) or macrometastatic (MAC). Presence of isolated tumor cells (ITCs) was also documented.

### Statistical analysis

Descriptive statistics were used to summarize the demographic and clinical characteristics of the cohort; absolute and relative frequencies for categorical variables were applied. For each category assessment, the final pathological finding was compared with the outcome as assessed by the imaging test.

The accuracy (or concordance) of MRI and ultrasound examination in assessing tumor size was evaluated in terms of discrepancy (< 10 mm *vs* ≥ 10 mm) between imaging and pathological finding. Accuracy was calculated separately for ultrasound and MRI measurements. Statistical comparison of the accuracy between ultrasound and MRI examinations was performed using the chi‐square test to determine any statistically significant differences.

Negative predictive value (NPV) was defined as the ratio of patients with truly negative imaging (MRI or ultrasound) results to the total population. Comparative analyses of the NPV for ultrasound and MRI were conducted for both parameters (parametrial invasion and lymph node involvement). The NPV of each imaging modality was calculated and compared using the chi‐square test to determine any statistically significant differences. Two‐sided α = 0.05 was adopted as the level of statistical significance. All analyses were performed using SPSS version 25.0.0.1, 2019 (IBM Corp., Armonk, NY, USA).

## RESULTS

### Cohort characteristics

A total of 816 patients who fulfilled the preoperative inclusion criteria, based on preoperative staging by MRI or ultrasound examination, and were scheduled for curative treatment, were preregistered in the SENTIX study. After excluding 126 patients for the reasons indicated in Figure 1, 690 patients were included in the *post‐hoc* analysis of the presented study.

Of the 690 patients, the majority had preoperative Stage IB1 (345 (50.0%)), and 536 (77.7%) underwent radical hysterectomy. Ultrasound and MRI examinations were performed as a single imaging test in 298 (43.2%) and 322 (46.7%) patients, respectively, while 70 (10.1%) patients underwent both imaging tests. All patients had negative parametrial involvement and non‐suspicious lymph nodes based on the preoperative clinical staging. The clinicopathological characteristics of all included patients are presented in Table 1.

**Table 1:** Clinicopathological characteristics of study cohort, overall and according to preoperative imaging method

Characteristic	All patients (*n* = 690)	MRI only (*n* = 322)	Ultrasound only (*n* = 298)	MRI + ultrasound (*n* = 70)
Age				
≤ 40 years	285 (41.3)	136 (42.2)	123 (41.3)	26 (37.1)
41–60 years	320 (46.4)	153 (47.5)	133 (44.6)	34 (48.6)
≥ 61 years	85 (12.3)	33 (10.2)	42 (14.1)	10 (14.3)
BMI				
≤ 25 kg/m^2^	395 (57.2)	192 (59.6)	164 (55.0)	39 (55.7)
25–29.9 kg/m^2^	162 (23.5)	76 (23.6)	68 (22.8)	18 (25.7)
≥ 30 kg/m^2^	133 (19.3)	54 (16.8)	66 (22.1)	13 (18.6)
ECOG‐PS				
0	664 (96.2)	312 (96.9)	285 (95.6)	67 (95.7)
1	26 (3.8)	10 (3.1)	13 (4.4)	3 (4.3)
2	0 (0)	0 (0)	0 (0)	0 (0)
Diagnostic procedure*				
Biopsy	294 (42.6)	140 (43.5)	125 (41.9)	41 (58.6)
Conization	418 (60.6)	188 (58.4)	176 (59.1)	30 (42.9)
Trachelectomy	2 (0.3)	0 (0)	2 (0.7)	0 (0)
Tumor stage (preoperative)				
IA1 + LVSI	36 (5.2)	15 (4.7)	19 (6.4)	2 (2.9)
IA2	70 (10.1)	37 (11.5)	31 (10.4)	2 (2.9)
IB1	345 (50.0)	166 (51.6)	142 (47.7)	37 (52.9)
IB2	239 (34.6)	104 (32.3)	106 (35.6)	29 (41.4)
Tumor type				
Squamous cell carcinoma	480 (69.6)	212 (65.8)	221 (74.2)	47 (67.1)
Adenocarcinoma HPV‐related	202 (29.3)	106 (32.9)	73 (24.5)	23 (32.9)
Adenosquamous carcinoma	7 (1.0)	4 (1.2)	3 (1.0)	0 (0)
NA	1 (0.1)	0 (0)	1 (0.3)	0 (0)
Maximum tumor size (preoperative)				
< 2 cm	447 (64.8)	215 (66.8)	191 (64.1)	41 (58.6)
2–4 cm	242 (35.1)	106 (32.9)	107 (35.9)	29 (41.4)
NA	1 (0.1)	1 (0.3)	0 (0)	0 (0)
Parametrectomy type				
B	171 (24.8)	113 (35.1)	42 (14.1)	16 (22.9)
C1	259 (37.5)	86 (26.7)	133 (44.6)	40 (57.1)
C2	106 (15.4)	59 (18.3)	41 (13.8)	6 (8.6)
NA/not done	154 (22.3)	64 (19.9)	82 (27.5)	8 (11.4)
Lymph node status (final pathology)				
MAC	41 (5.9)	19 (5.9)	18 (6.0)	4 (5.7)
MIC	37 (5.4)	17 (5.3)	13 (4.4)	7 (10.0)
ITC	23 (3.3)	6 (1.9)	16 (5.4)	1 (1.4)
Negative	589 (85.4)	280 (87.0)	251 (84.2)	58 (82.9)
Parametrial involvement (final pathology)†				
No	510/536 (95.1)	243/258 (94.2)	207/216 (95.8)	60/62 (96.8)
Yes	26/536 (4.9)	15/258 (5.8)	9/216 (4.2)	2/62 (3.2)‡
Vaginal involvement (final pathology)				
Yes	14 (2.0)	11 (3.4)	3 (1.0)	0 (0)
No	605 (87.7)	292 (90.7)	249 (83.6)	64 (91.4)
NA/not done	71 (10.3)	19 (5.9)	46 (15.4)	6 (8.6)
Maximum tumor size (final pathology)				
< 2 cm	438 (63.5)	208 (64.6)	192 (64.4)	38 (54.3)
2–4 cm	224 (32.5)	104 (32.3)	93 (31.2)	27 (38.6)
> 4 cm	28 (4.1)	10 (3.1)	13 (4.4)	5 (7.1)
Median (mm)	15.0 (1.0–40.0)	15.0 (1.5–38.0)	14.0 (0.1–40.0)	17.0 (5.2–41.6)

Data are given as *n* (%), *n*/*N* (%) or median (95% CI).

* In 24 patients (3.5%), both biopsy and conization were performed.

† Only if performed.

‡ One patient enrolled as a protocol deviation due to parametrial infiltration detected preoperatively on imaging.

BMI, body mass index; ECOG‐PS, Eastern Cooperative Oncology Group performance status; HPV, human papillomavirus; ITC, isolated tumor cells; LVSI, lymphovascular space invasion; MAC, macrometastasis; MIC, micrometastasis; MRI, magnetic resonance imaging; NA, not available.

### Tumor size assessment

For tumor size assessment, the maximum pathological size was compared with the maximum tumor size measured in the imaging test. The data from all 690 patients were included in the analysis.

**Figure 2:**
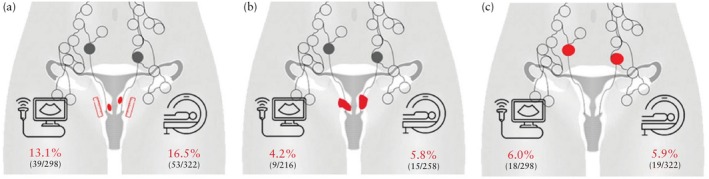
Illustrations depicting ultrasound examination and magnetic resonance imaging methods. Percentage of patients with (a) incorrect preoperative assessment of tumor size (undermeasured by ≥ 10 mm), (b) failed detection of parametrial invasion and (c) macrometastatic lymph node involvement are shown.

The difference in tumor size between imaging and pathology was < 10.0 mm in 585 (84.8%) patients, 10–19.9 mm in 76 (11.0%) patients and ≥ 20 mm in 29 (4.2%) patients (Figure 2, Table 2). Overall, a postoperative tumor size increase ≥ 10 mm was observed in 13.1% (39/298) and 16.5% (53/322) of patients after staging on ultrasound and MRI scans, respectively, with no significant difference in the accuracy of tumor measurement between the two imaging methods (*P* = 0.452).

**Table 2:** Preoperative diagnostic performance of ultrasound and magnetic resonance imaging (MRI) for assessment of parametrial invasion, lymph node (LN) involvement and tumor size

Parameter	Ultrasound only	MRI only	*P* *	MRI + ultrasound
Parametrial tumor invasion (confirmed by final pathology)†			0.624	
Yes	9/216 (4.2)	15/258 (5.8)		2/62 (3.2)§
No	207/216 (95.8)	243/258 (94.2)		60/62 (96.8)
Macrometastatic LN involvement (confirmed by final pathology)			0.876	
Yes	18/298 (6.0)	19/322 (5.9)		4/70 (5.7)
No	280/298 (94.0)	303/322 (94.1)		66/70 (94.3)
Discrepancy in tumor size‡			0.452	
< 5 mm	219/298 (73.5)	235/322 (73.0)		49/70 (70.0)
5–9.9 mm	40/298 (13.4)	34/322 (10.6)		8/70 (11.4)
10–19.9 mm	26/298 (8.7)	41/322 (12.7)		9/70 (12.9)
≥ 20 mm	13/298 (4.4)	12/322 (3.7)		4/70 (5.7)

Data are given as *n*/*N* (%).

* Comparison between ultrasound only and MRI only.

† Only patients with completed surgery included.

‡ Difference in maximum tumor size between preoperative imaging and pathology.

§ One patient enrolled as a protocol deviation due to parametrial infiltration detected preoperatively on imaging.

### Parametrial involvement

In the analysis of parametrial involvement, we used data from 536 patients in whom parametrectomy was performed, with data from ultrasound, MRI and both methods available in 216, 258 and 62 patients, respectively. One patient was excluded due to a protocol deviation because parametrial infiltration was detected preoperatively on imaging, thus the patient should not have been enrolled in the study. Based on the final pathological results, parametrial involvement was found in 25 patients, the majority of whom had a postoperative tumor size of 2–4 cm (16/25) and 6/25 had a tumor > 4 cm in size. Of the 25 patients, the depth of parametrial invasion was 1–2.5 mm in nine patients and ≥ 5 mm in five; the size of invasion was not available in seven and was not measurable (microscopic) in four. Ultrasound examination and MRI failed to detect parametrial involvement in nine (4.2%) and 15 (5.8%) patients (Figure 2, Table 2), respectively, with corresponding NPVs of 97.0% (95% CI, 0.95–0.99%) and 95.3% (95% CI, 0.93–0.98%), respectively. No significant difference in NPV between the two imaging methods was observed (*P* = 0.624).

### Lymph node involvement

All patients included in the trial underwent surgical lymph node staging. Lymph node involvement of any size was found in 101 (14.6%) patients in the study cohort; for all these patients, the preoperative imaging showed negative lymph nodes. The largest extent of metastasis in the SLN was classified as ITC, MIC and MAC in 23 (3.3%), 37 (5.4%) and 41 (5.9%) patients, respectively (Table S1). MAC was found in 18 (6.0%) patients who underwent preoperative staging with ultrasound examination and in 19 (5.9%) who underwent MRI (Figure 2, Table 2), with corresponding NPVs of 94.0% (95% CI, 0.91–0.97%) and 94.1% (95% CI, 0.92–0.97%), respectively. No significant difference in NPV between the two imaging methods was observed (*P* = 0.876).

## DISCUSSION

In this *post‐hoc* analysis of data from the multicenter SENTIX study including 690 prospectively enrolled patients with early‐stage cervical cancer, we demonstrated that ultrasound examination is comparable with MRI in assessing key parameters evaluated routinely in pelvic clinical staging. Notably, both modalities showed similar accuracy in tumor size measurement and comparable NPVs in detecting parametrial invasion and MAC lymph node involvement. Both methods were highly reliable for predicting the absence of parametrial involvement (NPV > 95%), though both showed similar lower NPVs for lymph node staging, by missing N1 status (metastases > 0.2 mm) in 11.3% of patients. Furthermore, both imaging modalities tended to underestimate tumor size (by ≥ 10 mm in approximately 15% of patients).

In its early stages, cervical cancer is characterized by local spread by direct invasion into the adjacent tissue or via lymphatic channels. Distant hematogenous metastases in tumors without lymph node involvement are therefore extremely rare. This explains why pretreatment pelvic staging is vital in triaging patients for further clinical management. According to joint European guidelines, MRI or ultrasound examination of the pelvis is recommended for pelvic staging^8^, with additional imaging tests such as positron emission tomography performed only secondarily to exclude distant spread in locally advanced tumors and/or in those with lymph node involvement^9^.

The use of ultrasound examination for clinical staging of cervical cancer has been debated since the 1990s^20^, with the feasibility and reliability of ultrasound examination often being challenged because of its dependency on special expertise, which is limited to a few sites only, or the lack of data on ultrasound examination staging of pelvic lymph nodes^25^. We are aware of only one international prospective multicenter study that enrolled 182 patients (FIGO Stages IA2–IIA) across four European sites, all with extensive expertise of ultrasound imaging in gynecological cancer, in which the authors concluded that the agreement between histology and ultrasound examination was significantly better for assessing residual tumor after cone biopsy (*P* < 0.001) and for parametrial invasion (*P* < 0.001) compared with the results obtained by MRI^15^.

Those findings, as well as the findings of this study, are very encouraging because ultrasound examination has several advantages over MRI, not just the much lower cost, which makes it an attractive imaging alternative, even in regions with good MRI availability. Ultrasound examination can be performed in a shorter timeframe than MRI, is widely available in gynecological clinics, does not require patient preparation and lacks contraindications. In routine clinical practice, ultrasound examination can be performed using the all‐in‐one approach, i.e. by one team involved in planning and performing surgery, allowing for more effective and tailored management.

MRI, on the other hand, is recommended traditionally to assess the pelvic extent of the disease because of its excellent soft‐tissue contrast, although fasting and administration of antispasmodic agents are advised in the current European guidelines, and conventional two‐dimensional MRI is advised to be combined with diffusion‐weighted imaging to merge morphological and functional sequences^26^. MRI limitations are related to the longer timeframe required to perform the examination, noise, limited availability in some regions, higher costs, claustrophobia and contraindications regarding metal implants, for example.

The three main parameters that are assessed routinely in the pretreatment workup of cervical cancer (tumor size, parametrial involvement and lymph node involvement) are crucial for making decisions about clinical management. International clinical practice guidelines agree that surgery is a treatment of choice in the early stages of cervical cancer, and that definitive chemoradiotherapy is recommended for locally advanced disease^8^, ^27^. In addition, in the early stages, tumor size is an important determinant of the type of cervical procedure, the type of lymph node staging and options regarding fertility‐sparing treatment.

The threshold for deciding between simple and radical hysterectomy is currently a depth of invasion of 5 mm (T1a2/T1b1), which is expected to shift to a tumor size of < 2 cm and limited stromal invasion based on the outcome of the SHAPE trial^28^.

A prospective study of 182 patients showed comparable size assessment using MRI and ultrasound examinations for small and large tumors^15^. This is in agreement with this study, in which we found no significant difference in the accuracy of tumor size undermeasurement between these imaging methods in our group of 690 patients (*P* = 0.452).

Parametrial invasion was frequently the primary cause of staging inaccuracy in previous studies^11^, ^29^. Because parametrial positivity is considered a high‐risk prognostic factor, affected patients should be referred for definitive chemoradiation, instead of combining radical surgery and adjuvant radiotherapy. Three recent meta‐analyses of studies using histological results as a reference concluded that ultrasound examination and MRI had similar diagnostic performance for assessing parametrial infiltration in cervical cancer, with a pooled sensitivity of 62–78% and a specificity of 91–96%^30^, ^31^, ^32^. In this study, parametrial infiltration was found on pathology in only 25/536 patients (NPV, 95.3%) who underwent parametrectomy, with the proportion of parametrial‐positive cases (NPV of preoperative parametrial assessment) not significantly different between patients undergoing preoperative staging by ultrasound and MRI examination (*P* = 0.624).

As for parametrial involvement, surgical treatment is not recommended for lymph node‐positive patients and accurate preoperative lymph node staging could help avoid unnecessary surgical morbidity and direct patients to definitive chemoradiotherapy^33^. Previously, only a few studies focused on lymph node assessment by ultrasound examination; many of which did not perform SLN biopsy followed by ultrastaging^25^, ^31^, ^32^, ^34^. Two recently published meta‐analyses on ultrasound for detecting lymph node involvement reported a sensitivity of 43–52% and a specificity of 95–96%, compared with 57% and 93%, respectively, for MRI^31^, ^32^. In this study, we detected lymph node involvement in 101 patients, in which the largest size was ITC, MIC and MAC in 23 (3.3%), 37 (5.4%) and 41 (5.9%) patients, respectively. Notably, MRI and ultrasound examination showed comparable NPVs in the assessment of MAC in this study (*P* = 0.876).

Operator dependency is a critical factor when considering the reliability of an imaging method or comparing two methods. Although the accuracy of an ultrasound examination is undoubtedly influenced by the expertise of the operator performing it, MRI, despite being considered less operator‐dependent because of its standardized protocols, still requires a high level of skill for accurate interpretation, especially in complex cervical cancer cases. Both modalities, therefore, present unique challenges in ensuring consistent and reliable preoperative triage of patients with cervical cancer, and the choice between them may depend on the availability of skilled operators.

Recent studies have explored interobserver agreement and the impact of experience on ultrasound in gynecological cancers. Pálsdóttir *et al*.^25^ demonstrated similar ability of experienced and less experienced ultrasound examiners to correctly identify cervical cancer, but the experienced examiners were better at excluding parametrial invasion. Fischerova *et al*.^35^ evaluated interobserver agreement of lymph node assessment in patients with ovarian cancer. The probability of correct classification of 380 video clips ranged from 0.956 to 0.975 and was not affected by the level of ultrasound experience of the examiner. The likelihood of correct classification increased with image quality and diagnostic confidence, and it was affected by anatomical region.

One of the limitations of the current study is that the outcome applies only to the group of patients with early‐stage cervical cancer, so does not allow for full generalizability to other cohorts, as the included cohort had a low rate of parametrial and lymph node involvement. Although the fact that only one imaging method was required in each patient can be viewed as a limitation, we believe that this translates into an advantage. Each participating site was instructed to choose the method for which they had the greatest expertise, the method used in routine clinical practice or the method they considered to be most suitable for the individual patient. In previous studies in which both imaging modalities were required in each patient, investigators were forced to compare one method, which they used routinely and had good experience, with the second method, for which they had more limited expertise. The key strengths of this study are the size of the group, the multicenter setting and the standardization of procedures in a prospective study, including preoperative imaging and pathological assessment.

In conclusion, our *post‐hoc* analysis of data from patients who were prospectively enrolled in the SENTIX trial has shown that ultrasound examination is comparable with MRI in the pretreatment assessment of all three clinically relevant parameters for the management of patients with early‐stage cervical cancer, namely tumor size assessment and the assessment of parametrial invasion and lymph node involvement. Ultrasound examination should be considered as part of preoperative pelvic staging in early‐stage cervical cancer, especially in limited‐resource regions where MRI is unavailable. Both methods tended to underestimate the tumor size and missed N1 lymph node metastases in 11.3% of patients.

## Supporting information


**Table S1** Lymph node involvement in 690 patients with early‐stage cervical cancer, according to postoperative stage

## Data Availability

Data will be provided by the corresponding author upon reasonable request.
