# Supplementary material for: Magnetic resonance imaging and ultrasound examination in preoperative pelvic staging of early‐stage cervical cancer: post‐hoc analysis of SENTIX study
Source: Ultrasound Obstet Gynecol. 2025 Mar 25;65(4):495–502. doi: 10.1002/uog.29205 (PMC11961100; doi:10.1002/uog.29205)
Supplement: Supplementary file 1 — Table S1 Lymph node involvement in 690 patients with early‐stage cervical cancer, according to postoperative stage [file UOG-65-495-s001.docx]

**TABLE S1** Lymph node involvement in 690 patients with early-stage cervical cancer, according to postoperative stage

| LN involvement |  |  | N0 | N1 | MAC | MIC | ITC |
| --- | --- | --- | --- | --- | --- | --- | --- |
| Postoperative stage (p) | **1a1+LVSI** | **n = 44** | 42 (95.45%) | 1 (2.27%) | 1 (2.27%) | 0 (0.00%) | 1 (2.27%) |
|  | **1a2** | **n = 36** | 34 (94.44%) | 2 (5.56%) | 2 (5.56%) | 0 (0.00%) | 0 (0.00%) |
|  | **1b1** | **n = 337** | 313 (92.88%) | 16 (4.75%) | 9 (2.67%) | 7 (2.08%) | 8 (2.37%) |
|  | **1b2** | **n = 200** | 152 (76.00%) | 35 (17.5%) | 16 (8.00%) | 19 (9.50%) | 13 (6.50%) |
|  | **≥1b3** | **n = 73** | 48 (65.75%) | 24 (32.90%) | 13 (17.81%) | 11 (15.07%) | 1 (1.37%) |
| All patients | | **n = 690** | 589 (85.36%) | 78 (11.30%) | 41 (5.94%) | 37 (5.36%) | 23 (3.33%) |

Values are no. of patients (%)

Abbreviations: ITC, isolated tumor cells; LVSI, lymphovascular space invasion; MAC, macrometastasis; MIC, micrometastasis; N1, MIC+MAC.
